# Perceptions about Research Participation among Individuals at Risk and Individuals with Premanifest Huntington’s Disease: A Survey Conducted by the European Huntington Association

**DOI:** 10.3390/jpm11080815

**Published:** 2021-08-20

**Authors:** Filipa Júlio, Ruth Blanco, Josè Perez Casanova, Barbara D’Alessio, Beatrice De Schepper, Dina De Sousa, Paul De Sousa, Cristina Ferreira, Hans Gommans, Rob Haselberg, Emilie Hermant, Danuta Lis, Sabrina Maffi, Svein Olaf Olsen, Marios Papantoniou, Ferdinando Squitieri, Marina Tretyakova, Zaynab Umakhanova, Vladimír Václavík, Michaela Winkelmann, Astri Arnesen, on behalf of the European Huntington Association

**Affiliations:** 1 European Huntington Association, Spelonckvaart 30, 9180 Moerbeke (Waas), Belgium; contacto@e-huntington.es (R.B.); joseperezcasanova@yahoo.es (J.P.C.); barbara.dalessio@lirh.it (B.D.); bea@eurohuntington.org (B.D.S.); dina@eurohuntington.org (D.D.S.); Paul.deSousa@ed.ac.uk (P.D.S.); scpsenas@gmail.com (C.F.); jhmgommans@hotmail.nl (H.G.); Rob@huntington.nl (R.H.); emiliehermant@gmail.com (E.H.); d.lis@huntington.pl (D.L.); svein@eurohuntington.org (S.O.O.); marios_papantoniou@hotmail.com (M.P.); mntretyakova@orphanpeople.ru (M.T.); zumakhanova@gmail.com (Z.U.); huntingtonovachoroba@gmail.com (V.V.); m.winkelmann@dhh-ev.de (M.W.); astri@eurohuntington.org (A.A.); 2 Associação Portuguesa dos Doentes de Huntington, Praça António Baião, nº. 7 loja A, 1500-567 Lisboa, Portugal; 3 Asociación Corea de Huntington Española, Avenida Cardenal Herrera Oria 80B, C.P. 28034 Madrid, Spain; 4 Fondazione Lega Italiana Ricerca Huntington, Via Varese 31, 00185 Rome, Italy; 5 Huntington Liga, Spelonckvaart 30, 9180 Moerbeke (Waas), Belgium; 6 Scottish Huntington’s Association, Business First, Linwood Point, Paisley PA1 2FB, UK; 7 Vereniging Van Huntington, Stationsplein 125, 3818 LE Amersfoort, The Netherlands; 8 L’Association Huntington France, 44, rue du Château des Rentiers, 75013 Paris, France; 9 Polskie Stowarzyszenie Choroby Huntingtona, Rękodzielnicza 17 A, 02-267 Warszawa, Poland; 10 Huntington and Rare Diseases Unit, IRCCS Casa Sollievo della Sofferenza Research Hospital, San Giovanni Rotondo, 71013 Foggia, Italy; sabrina.maffi@gmail.com (S.M.); ferdinandosquitieri@yahoo.it (F.S.); 11 Landsforeningen for Huntingtons sykdom, LHS v/Geir Viksund, Grønebrekka 32, 5306 Askøy, Norway; 12 Huntington’s Disease Association of Cyprus, 89, Arch. Makarios III Avenue, Limassol 3020, Cyprus; 13 Orphan People, Cпapтaкoвcкaя yлицa, дoм 9, cтpoeниe 1, 105 066 Moscow, Russia; 14 Spoločnost’ Pre Pomoc Pri Huntingtonovej Chorobe, Gagarinova 979, 900 61 Gajary, Slovakia; 15 Deutsche Huntington Hilfe E.V., Geschaefts und Beratungsstelle Falkstrasse 73-77, D 47058 Duisburg, Germany

**Keywords:** Huntington’s disease, premanifest Huntington’s disease, clinical research, clinical trials, survey

## Abstract

There has been great progress in Huntington’s disease (HD) research. Yet, effective treatments to halt disease before the onset of disabling symptoms are still unavailable. Scientific breakthroughs require an active and lasting commitment from families. However, they are traditionally less involved and heard in studies. Accordingly, the European Huntington Association (EHA) surveyed individuals at risk (HDRisk) and with premanifest HD (PreHD) to determine which factors affect their willingness to participate in research. Questions assessed research experience and knowledge, information sources, reasons for involvement and noninvolvement, and factors preventing and facilitating participation. The survey included 525 individuals, of which 68.8% never participated in studies and 38.6% reported limited research knowledge. Furthermore, 52% trusted patient organizations to get research information. Reasons for involvement were altruistic and more important than reasons for noninvolvement, which were related to negative emotions. Obstacles included time/financial constraints and invasive procedures, while professional support was seen as a facilitator. PreHD individuals reported less obstacles to research participation than HDRisk individuals. Overall, a high motivation to participate in research was noted, despite limited experience and literacy. This motivation is influenced by subjective and objective factors and, importantly, by HD status. Patient organizations have a key role in fostering motivation through education and support.

## 1. Introduction

Over the last decade, there have been significant advances in Huntington’s disease (HD) research, which have culminated in several new therapeutic compounds either in clinical evaluation or on the verge of being so [1,2]. However, effective disease-modifying therapies that can change or stop the relentless disease course before the onset of any disabling symptoms are still lacking [3].

Since 2016, the European Huntington Association (EHA) has focused on increasing the awareness of the European HD community about the importance of supporting and participating in research. One of the key messages has been that successful treatments for HD cannot be developed without an active and long-lasting commitment from patients and families. In this context, the EHA has been involved in the design and implementation of several initiatives aimed at broadening and improving the clinical research knowledge of the European HD community. These include the “Stronger Together” project [4], the HD TrialFinder platform [5], the HD-COPE taskforce [6,7], and the organization of regular meetings between families and professionals, both at national and at international levels. The EHA believes that these initiatives helped boost the number of participants in large multicenter observational studies such as Enroll-HD [8,9]. This study resource and the willingness of those enrolled to participate in research has attracted pharmaceutical companies to HD. In fact, while being a rare disease, HD may help to develop therapeutic compounds or strategies applicable in time to more prevalent diseases. It has also underpinned the rapid pace and dissemination of encouraging preliminary findings of some studies, such as Generation HD1 [10,11]. Several ongoing clinical trials for new therapeutic interventions for HD are anticipated to be conducted within the next 5 years. Many of these have had input from representatives of the EHA member associations from their very beginning (e.g., [12,13,14,15]). 

Multiple ongoing clinical trials create a challenge for rare diseases such as HD, where the baseline number of individuals that can potentially participate in each study is already small by default. As finding enough of the right people to join is the biggest challenge of any clinical study or trial [9], and as the lack of participants in clinical trials is the number one obstacle to success [16], having concurrent HD trials requiring a large number of participants may threaten the recruitment of a sufficient study sample and the capacity to complete enrollment in a timely manner [17]. Moreover, critically, the participation in one specific trial will, in most cases, exclude the possibility of being involved in other trials [18]. Furthermore, there is an exponential interest and need for individuals in a premanifest/prodromal HD stage to participate in research studies, as one of the major goals for interventional therapy in HD is preventive treatment prior to the onset of disabling symptoms [19]. However, most individuals at risk for HD (>80%) do not undergo predictive genetic testing [20], and there are reports of a decline in the rate of predictive test uptake over time, as fewer people get tested now [21]. This can jeopardize the design of transparent trials for premanifest HD research—standard randomized controlled trials where asymptomatic individuals with a known positive genetic test result for HD receive either experimental treatment or placebo—as only those with known genetic status will be able to participate [22]. More importantly, because individuals at risk for HD and individuals in a premanifest/prodromal HD stage do not experience obvious clinical symptoms and do not see themselves as patients [23,24], they have little contact with the healthcare system in relation to HD, which may cause them to miss important research opportunities. All these issues strengthen the need for internationally validated guidelines to address the complexities of predictive genetic testing and counselling and of the transparent enrollment of individuals in a premanifest/prodromal HD stage in clinical studies [22,25,26].

Willingness to participate in research can be adversely impacted by inevitable setbacks in research and clinical trials, such as the premature ending of trials which fail to demonstrate benefit. When these occur, trial sponsors have a duty to properly explain why. The failure of a trial can only compound disillusionment and might influence future study engagement. More prevalent neurodegenerative diseases such as Alzheimer’s disease (AD) and Parkinson’s disease (PD) have created taskforces to deal with patient engagement in clinical trials. These tackle issues such as recruiting enough participants, maintaining participant retention, ensuring that recruits are representative of the specific clinical population, dealing with screening failures, and recruiting in preclinical disease [16,27,28]. The EHA believes such efforts make even more sense in rare clinical conditions such as HD, where recruitment and retention problems are likely to occur given the scarce number of potential trial participants.

To help HD families engage in research in a knowledgeable way, the EHA has recently launched a new project called “Moving Forward: Toward a Future with Effective Disease-Modifying Therapies for Huntington’s Disease” [29]. The aim of Moving Forward is to mobilize the European HD community to show a strong and long-term engagement with research. The EHA wants to ensure there will be a continuous flow of individuals signing up to take part in studies and trials, thereby ensuring there are no delays in HD clinical progress due to the slow recruitment of potential study participants. Specifically, this project targets individuals that are traditionally less involved with research—those at risk for HD whose genetic status is unknown (HDRisk) and those that tested positive for HD and are in a so-called premanifest or prodromal disease stage (PreHD). Moving Forward has five central goals: increase trial awareness, increase health literacy, reduce barriers to study participation, build up the research staff skills on ways to communicate and relate with HD families, and increase clinical trial readiness. Achieving these goals in the near future will guarantee that the community is ready when the first trials designed to address preclinical HD stages are launched and will bring patients and families closer to effective treatments. 

To understand the current situation and create a good knowledge base to plan meaningful actions within the Moving Forward project, the EHA has developed a survey to assess the perceptions and experiences about research participation among individuals with HDRisk and PreHD. The goal was to better recognize the needs, worries, and wishes of those usually less involved in HD clinical studies and trials. The EHA believes that the insights offered by this study will assist in bringing these groups closer to research sites and will provide a solid framework to address the recruitment and retention challenges posed by the different HD trials planned for the near future. Lastly, as a European umbrella organization, the EHA is in a unique position to coordinate and run multinational projects such as this one, helping to build bridges and facilitating the communication between all the key players and stakeholders of the research process in Huntington’s disease.

## 2. Materials and Methods

The EHA developed an anonymous online survey to collect information about the perceptions and experiences of research participation among individuals with HDRisk and PreHD across Europe (see Supplementary Material: the survey in Supplementary S1).

### 2.1. Participants

The study population was composed of a random sample of individuals who self-reported as being either at risk for HD (HDRisk) or in a premanifest HD stage (PreHD). 

The EHA wanted to capture the view of these two specific groups on research participation, as clinical trials to slow, reverse, or halt disease progression during the earliest period of disease are gradually on the horizon [30]. The information obtained through the online survey aims to better accommodate the specificities of these groups into trial design and implementation [19].

HDRisk individuals were defined as adult children of an HD patient who are in a prediagnostic phase, i.e., they do not know their gene status since they have chosen not to undergo the genetic testing process for HD [3,31], and they have not been clinically diagnosed as having HD [30]. Nevertheless, as Walker [31] noted, many HD experts know exuberant symptomatic patients who tell everyone they are only “at risk” for HD, so prediagnostic is not necessarily synonymous with presymptomatic.

PreHD individuals were defined as clinically unaffected adults that learned of their HD gene carrier status through a genetic test [3]. They have been genetically identified as carriers of a CAG repeat expansion of ≥36 repeats in the HD gene but have not yet received an HD clinical diagnosis [30]. They can be asymptomatic, show nonspecific signs and symptoms of HD, or have signs and symptoms sufficient for the diagnosis of HD even though the genetic diagnosis still has not been clinically confirmed [31]. These circumstances lead to a further division of the premanifest HD period into a “presymptomatic” phase, in which individuals show no subjective symptoms, measurable abnormalities, or clinical signs of HD, and a “prodromal” phase, during which there is a gradual appearance of subtle motor, cognitive, and behavioral changes that do not meet the criteria for formal HD diagnosis [32].

The EHA reached out to these two specific groups within the HD community using a multidimensional recruitment approach. Communication was made through the social media of the EHA and through the different national HD organizations e.g., mailing list, Facebook page, website, and newsletter. Since the EHA has 45 member associations representing 30 countries, we believe that a significant number of individuals were notified about the survey. To complement the survey dissemination and reinforce the inclusion criteria for study participation, the EHA prepared a short written introduction about the nature, goals, and target population of this work, which was also translated by member associations into their native languages when announcing this project to their community (see Supplementary Material: information about the survey in Supplementary S2). Additionally, the EHA included a short definition of HDRisk and PreHD in the survey question about HD status (see Supplementary Material: survey in Supplementary S1).

The study was conducted in compliance with the Declaration of Helsinki and the EU General Data Protection Regulation and was approved by the Institutional Review Board of Lega Italiana Ricerca Huntington (LIRH) Foundation (protocol code n.3 270421). 

### 2.2. Survey

The survey was created through the SurveyMonkey platform [33] and contained 12 questions. The entire survey took around 8 min to complete.

The aim of the first five questions was to gather demographic information, namely, the country, gender, age, education level, and HD status of survey respondents. A short description of the two target groups was provided under the HD status question, to reduce the unavoidable assessment bias related to self-report measures. The next two questions assessed the previous research experience of respondents and self-appraisal of their knowledge of HD research. Question number eight was a multiple-answer question, where respondents had to pick from a list all the sources they use to get information about HD research. On the following two questions, respondents had to rate on a Likert-type scale (ranging from “not important” to “very important”) the importance of reasons for getting involved and not getting involved in research. The last two questions allowed for multiple answers, and both required respondents to pick from a list all the factors they thought can prevent or facilitate research participation.

After the survey was conceived, five individuals from the target groups with different personal backgrounds were asked to fill in the survey and give us their suggestions about any changes or improvements to be made. Their feedback was accommodated into the final version of the survey. 

The survey was made available in 12 different languages translated by representatives from EHA member associations into their native languages, namely, Dutch (with variants for use in Belgium and The Netherlands), English, French, German, Italian, Norwegian, Polish, Portuguese, Russian, Slovakian, and Spanish. 

### 2.3. Statistical Analysis 

The statistical analysis had two successive steps. First, the data collected from all survey respondents were characterized in terms of frequency and percentage distributions. Next, responses were subdivided according to HD status to see if there were any statistically significant differences in the way individuals with HDRisk and PreHD answered the survey. This latter step provides critical information for the design and implementation of actions tailored to each specific HD group.

When examining the differences between the HDRisk and the PreHD groups, nominal/categorical variables were compared resorting to chi-square tests of independence. Comparisons of quantitative variables between the two groups were performed using Mann–Whitney *U* tests. The analysis was carried out using IBM SPSS Statistics 24, and all calculations adopted a significance level of *α* = 0.05.

## 3. Results

The survey was filled by 525 individuals from 27 countries: Andorra (0.19%), Australia (0.76%), Austria (0.38%), Belarus (0.19%), Belgium (4%), Ecuador (0.19%), Finland (0.76%), France (1.33%), Germany (10.67%), Ireland (3.43%), Italy (26.1%), Kazakhstan (0.19%), Malta (0.19%), Mexico (0.38%), The Netherlands (16.57%), New Zealand (0.57%), Norway (5.90%), Pakistan (0.19%), Poland (8.19%), Portugal (2.86%), Romania (0.19%), Russian Federation (6.67%), Slovakia (1.52%), Spain (4.76%), Sweden (0.19%), UK and Northern Ireland (3.05%), and the United States of America (0.57%). 

### 3.1. Demographics

The number of individuals with HDRisk and PreHD answering the survey was comparable (50.1% and 49.9%, respectively) (see Table 1).

Most of the respondents were women (71.6%) and were below 45 years of age (71.2%). The global education level was relatively high, as 78.7% of participants had at least graduated from high school. Most respondents had never participated in HD clinical studies and trials (68.8%).

When comparing the two HD groups, we found that the HDRisk group had significantly more individuals in the 18–24 age interval and fewer individuals in the 55–64 age interval than the PreHD group (*χ*^2^(1) = 10.631, *p* = 0.001, and *χ*^2^(1) = 4.920, *p* = 0.027, respectively). The PreHD group reported significantly more previous research experience than the HDRisk group (*χ*^2^(1) = 54.384, *p* < 0.001). These results indicate that individuals with HDRisk were younger and less familiar with HD research than individuals with PreHD.

### 3.2. Level of Knowledge and Sources of Information about HD Research

Although 28.6% of survey respondents considered that their knowledge about HD research was good, 38.6% considered that their knowledge was not good or should be better (see Table 2).

Respondents specified the internet as the main source to get information about HD research; 78.5% pointed to it as their way to get updated about studies and trials. More than half of the survey participants signaled HD associations and support groups as their source to get news about HD research (52%). Healthcare professionals (25.5%) and family members (25.3%) were equally identified as preferred sources for information about HD research. 

The group analysis showed that individuals with HDRisk reported significantly more than individuals with PreHD that their knowledge about HD research “should be better” (*χ*^2^(1) = 8.061, *p* = 0.005). The PreHD group considered more often that their research knowledge was “good” and “excellent” compared to the HDRisk group (*χ*^2^(1) = 7.467, *p* = 0.006 and *χ*^2^(1) = 6.015, *p* = 0.014, respectively). Additionally, we found that the HDRisk group relied significantly more on family members to get information about HD research than the PreHD group (*χ*^2^(1) = 8.321, *p* = 0.004). These results reveal that the PreHD group reported an increased knowledge about HD research compared to the HDRisk group, which depended more on family members to get information about HD studies and trials. 

### 3.3. Reasons for Involvement and Noninvolvement in Research

**Figure 1:**
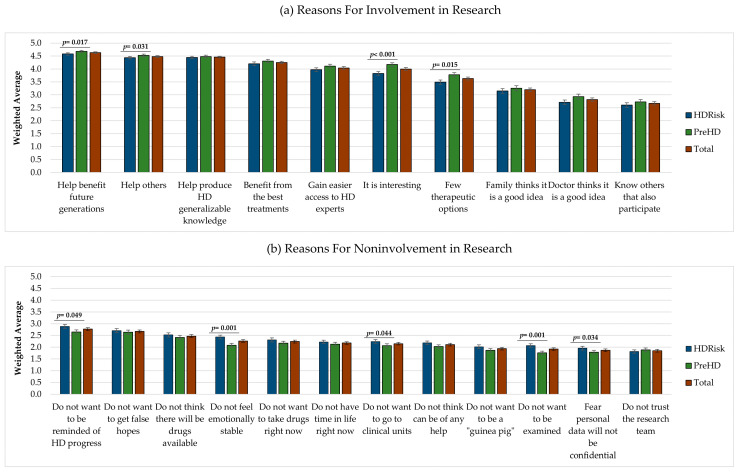
Reasons for involvement (**a**) and noninvolvement (**b**) in research (weighted average).

Respondents were asked to score the importance of reasons for involvement or noninvolvement in research on a scale ranging from 1 (not important) to 5 (very important). We observed that participants gave higher importance to the reasons for involvement than to the reasons for noninvolvement (see Figure 1).

Eight out of the 10 reasons for getting involved in research were rated above 3 and, thus, were considered, on average, moderately important, important, or very important (see Figure 1a). On the contrary, all 12 reasons for not getting involved in research were scored below 3 and, thus, were considered, on average, as not important or slightly important (see Figure 1b). The reasons for involvement rated with the highest importance were “I will help to benefit future generations” (*M* = 4.63, *SE* = 0.03), “I will help others” (*M* = 4.48, *SE* = 0.03), and “I will help to produce generalizable knowledge about HD” (*M* = 4.46, *SE* = 0.04). The reasons rated as more important for not getting involved in research were “I do not want to be reminded about the future progression of my disease” (*M* = 2.77, *SE* = 0.06), “I do not want to get false hopes” (*M* = 2.67, *SE* = 0.06), and “I do not think there will be any drugs available in my lifetime” (*M* = 2.47, *SE* = 0.06).

At a group level, we found statistically significant differences in the reasons selected by individuals with HDRisk and PreHD to get involved and not get involved in research. Overall, the PreHD group gave higher importance to the reasons for getting involved in HD research, and the HDRisk group gave higher importance to the reasons for not getting involved in research. Specifically, the PreHD group considered the following reasons to engage in research as more important than the HDRisk group: “I will help to benefit future generations” (*U* = 31196, *p* = 0.017), “I will help others” (*U* = 31225, *p* = 0.031), “I have few therapeutic options” (*U* = 30395, *p* = 0.015), and “I think it is interesting” (*U* = 28407, *p* < 0.001). On the contrary, the HDRisk considered some of the reasons for not getting involved in research as more important than the PreHD group, namely, “I do not want to be reminded about the future progression of my disease” (*U* = 31114, *p* = 0.049), “I do not want to go to clinical units” (*U* = 31144.5, *p* = 0.044), “I do not want to be examined” (*U* = 28959, *p* = 0.001), “I do not feel emotionally stable” (*U* = 28832.5, *p* = 0.001), and “I fear that my personal data will not be treated as confidential information” (*U* = 31231, *p* = 0.034). These results suggest that the PreHD group was more motivated to get involved in HD research than the HDRisk group, and that respondents with HDRisk displayed more emotional barriers for participating in studies.

### 3.4. Factors Influencing HD Research Participation

Regarding the factors that may facilitate or prevent participation in HD studies, respondents were asked to pick from two lists all the factors they thought could apply to them. 

The top three factors preventing research participation were “I would have personal expenses” (33.1%), “I would have to go through invasive procedures (e.g., lumbar puncture or surgery)” (31.4%), and “I would have to skip workdays/hours” (30.3%) (see Table 3).

The top three factors facilitating research participation were “I would gain easier access to treatments, health professionals, and HD experts” (61.7%), “I would have regular feedback about my health condition” (55.4%), and “I would have psychological and social care available” (50.7%). Several differences were found between the answers of the HDRisk and PreHD groups to these questions. A significantly higher percentage of individuals with HDRisk than individuals with PreHD selected the following preventing factors: “I would have to find someone to take care of my sick parent” (*χ*^2^(1) = 12.737, *p* < 0.001), “I would have to skip workdays/hours” (*χ*^2^(1) = 8.501, *p* = 0.004), “I would have to be involved in the study for a long time” (*χ*^2^(1) = 10.519, *p* = 0.001), “I would have to go through invasive procedures (e.g., lumbar puncture or surgery)” (*χ*^2^(1) = 7.273, *p* = 0.007), “I would have to do cognitive assessments” (*χ*^2^(1) = 3.982, *p* = 0.046), and “I would have to rethink my decisions about family planning” (*χ*^2^(1) = 20.653, *p* < 0.001). More individuals from the PreHD group than from the HDRisk chose the answer “none of the above” to this question (*χ*^2^(1) = 8.561, *p* = 0.003). A higher number of individuals with HDRisk than individuals with PreHD signaled the following facilitating factors: “I would have psychological and social care available” (*χ*^2^(1) = 4.953, *p* = 0.026), and “I would not have to go through invasive procedures (e.g., lumbar puncture or surgery)” (*χ*^2^(1) = 6.994, *p* = 0.008). Taken together, these results suggest that respondents with PreHD found fewer obstacles to research participation than respondents with HDRisk, who seemed to need more support to get engaged in studies.

## 4. Discussion

This study offers new and comprehensive insights into the topic of research participation in HD, and it is the first to focus exclusively on purportedly preclinical HD groups. First, we showed that individuals with HDRisk and PreHD report high motivation to take part in studies, despite having limited research experience and literacy. Second, we found that this motivation to participate in research is influenced by both personal factors, such as psychological, familial, and financial stability, and interpersonal factors, such as access and support from health professionals. Third, we showed that significant differences about research participation emerge according to HD status; individuals with PreHD report increased experience and higher motivation to take part in studies and consider themselves more knowledgeable than individuals with HDRisk. Lastly, we validated the key role of HD associations in fostering research engagement through the education and support of groups traditionally less involved in studies and trials.

### 4.1. HD Research Experience and Literacy 

Regarding research experience, there were two main findings: on the one hand, most respondents never participated in HD research; on the other hand, HD research experience is closely linked to HD status, whereby more individuals with PreHD than with HDRisk reported prior study participation. This latter finding is relevant as individuals with previous research experience have fewer negative expectations about trials than those without research experience, who may have more concerns to address when discussing trial opportunities [34], and also because it reinforces the value of experiential knowledge over scientific knowledge [35]. Thus, these findings suggest that individuals with PreHD might be helpful in promoting the engagement of individuals with HDRisk in studies and trials, by sharing their research experience and answering all the questions that usually arise prior to study participation.

Regarding research knowledge, our work corroborates previous findings about limited research literacy in HD families [36]. Notably, we also demonstrated that the level of research knowledge differs according to HD status; individuals with PreHD reported better knowledge than individuals with HDRisk. Since the willingness to participate in research has proven to be positively correlated with having adequate knowledge about clinical trials [37], educational actions targeting these HD groups, particularly individuals with HDRisk, might be useful to increase trial adherence. 

Regarding the sources to get research information, we made several important discoveries. First, both groups mainly look online for research news and updates. However, since it may be difficult to sort through, prioritize, and determine the value of the content that one can find online [38], this result is somewhat equivocal, as guidance to navigate and understand that information is not always available. Second, and importantly, HD associations and support groups were seen as trusted sources of information by the majority of survey respondents. This finding validates the crucial role of patient organizations in incrementing research education and participation [17,36] and providing access to research information [38,39]. Furthermore, it gives the lay associations an increased responsibility to reduce misinformation and pass on the right message to HD families. Third, since both family members and clinicians were identified as reliable informants for HD studies and trials, there is a need to increase research literacy not only among HD families, but also among health professionals, particularly those less acquainted with HD. A study done in AD found that most primary care doctors are not aware of the trials taking place in their area, and that this can pose a problem to the families that depend on them to have information about ongoing studies [28]. To prevent this from happening in HD, information should be disseminated across all those directly and indirectly involved in the research process. Lastly, as our results show that individuals with HDRisk resort significantly more to family members for information than individuals with PreHD, and since family encouragement can influence the decision to take part in studies and trials [40], the extended family should be mobilized to improve the research engagement of individuals with HDRisk. 

### 4.2. Motivators for HD Research Participation

Overall, survey respondents showed great motivation to take part in studies and trials, which goes in line with previous work that also found great interest in clinical trial participation among the HD population [34,36,41]. Notably, we found that the motivators for research participation differ according to HD status, which suggests that different approaches should be adopted to reach out for individuals with HDRisk and PreHD. Specifically, research contacts may be more effective if they enhance the emotional stability of the HDRisk group and address the therapeutic misconceptions of the PreHD group. Thus, we demonstrated that providing distinct psychological support and education about the research process to these specific groups within the HD community is crucial to increase their commitment to studies and trials.

#### 4.2.1. Reasons for Involvement in Research

The reasons considered as most important for getting involved in research were all quite altruistic and focused on what this can bring to others. Altruism has previously been reported as one of the key reasons to participate in studies and trials, not only in HD [42], but also in other neurological conditions, such as PD, where 90% out of 680 PD patients would participate in studies to help other patients [37]. Nevertheless, we found that the possibility of benefiting from treatments is also a strong reason for getting involved in studies and trials. This failure to understand that the primary purpose of research is to produce generalizable knowledge and not personal benefits is called therapeutic misconception [43,44,45], which is a common finding in neurodegenerative diseases. Bardakjian et al. [41] showed that the prospect of improving one’s own quality of life is an important motivating factor for trial participation in the HD community; Cotter et al. [34] found that HD patients agreed with statements that pointed personal benefit as the main reason for research participation; de Melo-Martín et al. [38] summarized the view of PD patients about clinical trial participation in the statement “I hope it helps me, but I’m doing it for others”; Grace Cannard et al. [40] found that PD patients indicate the desire to receive the best medical treatment as one of their primary motivations for enrolling in trials; lastly, Reijula et al. [37] concluded that almost 80% out of 680 PD patients think that clinical trials are aimed at finding the best treatment for them. Since therapeutic misconception can jeopardize the transparency of research participation [22,34], we believe this is a topic that should be addressed by researchers, clinicians, and patient advocates when presenting study opportunities to individuals with HDRisk and PreHD. 

#### 4.2.2. Reasons for Noninvolvement in Research

The reasons rated as most important for not getting involved in research were related to negative emotions, namely, a sense of hopelessness and anxiety about the future, which proves the relevance of having psychological care available to these specific HD groups, so that these negative emotions and thoughts can be overcome [46]. Furthermore, these results provide important indications about relevant topics to tackle when getting in touch with potential research candidates from both groups.

#### 4.2.3. Group-Specific Motivators of Research Participation

Looking closer at research involvement according to HD status, we found that respondents with PreHD considered altruism, lack of therapeutic options, and personal interest as more important than respondents with HDRisk. Previously, Cotter et al. [34] demonstrated that the risk of therapeutic misconception in HD is high because of the lack of alternative treatments. Cleret de Langavant et al. [42] also showed that the absence of curative treatment for HD may affect the decision process of potential study participants, leaving them more susceptible to coercion, as they have few therapeutic options. Hence, we believe that this finding further reinforces the importance of having researchers, clinicians, and patient representatives addressing therapeutic misconception when approaching these groups to participate in research, particularly individuals with PreHD [22]. Examining the group differences regarding the reasons for noninvolvement in research, we found that individuals with HDRisk considered emotional stability and confidentiality as more important than individuals with PreHD. These findings reinforce previous work that showed increased psychopathology [47] and frequent worrying about eventual confidentiality breaks [48] in individuals at risk for HD. Furthermore, the reasons underlying the noninvolvement of individuals with HDRisk in research seem to match the reasons underlying their decision not to undergo genetic testing, namely, the lack of an effective treatment for the disease and the emotional burden of this situation [49]. Therefore, it may be useful to debate research participation in the context of genetic counseling with individuals with HDRisk.

### 4.3. Moderators of HD Research Participation

On the factors that can influence research participation, survey respondents picked more facilitators than barriers to engage in studies. We found that facilitators include having easier access and more support from health professionals and barriers are related to tangible issues, such as time and financial constraints or study procedures. Interestingly, our work demonstrated that the willingness to participate in research is strongly influenced by HD status; individuals with HDRisk indicated more barriers to participation and needed more support to engage in studies than individuals with PreHD. Thus, these findings highlight the relevance of planning tailored interventions to better fit the specific needs of each of these HD groups and promote their meaningful commitment to the research process.

#### 4.3.1. Factors Facilitating Research Participation

Our results indicate that research participation is facilitated by having an easier access to HD experts and treatments and getting more support from health professionals. This finding consubstantiates recent reports. In HD, Davies et al. [50] showed that having a good relationship and a regular communication with the study team, and improving the access to the study site would encourage participation in the Enroll-HD study [8,9]; in PD, de Melo-Martín et al. [35] demonstrated the relevance of building trusting relationships to facilitate research participation; in AD, Boada et al. [28] concluded that improving communication, offering regular feedback, and building trustful relationships are key factors to increase trial engagement and retention. Hence, similarly to what happens in other neurodegenerative conditions, enhancing the communication and trust between potential research participants and health professionals and providing continuous support to individuals with HDRisk and PreHD seem to be critical to increase research adherence and ensure that the specific needs of these groups are being understood and met [28]. 

#### 4.3.2. Factors Preventing Research Participation

Our work showed that practical issues such as time and financial constraints, as well as the invasiveness of the study methodology, were seen as significant barriers to research participation by most survey respondents. This finding is in line with previous studies, specifically, with Goodman et al. [36], who showed that the time missed from work was one great obstacle to HD clinical trial participation, and that compensatory measures such as reimbursing travel expenses and having Saturday assessments were strong incentives to reverse this situation. Prior work done in HD also found that the willingness to participate in research decreases as the invasiveness of procedures increases [34,41]. In PD, fear of surgery, travel expenses, and time off work were found to be significant barriers to patient recruitment in trials [40]. In AD, trial participation was proved to be more daunting for patients who travel great distances [51]. Thus, the factors that hinder HDRisk and PreHD research participation seem to be easily addressable with some flexibility or simple arrangements from the research team, e.g., reimbursing the traveling costs of study participants or adjusting the assessment schedule to better fit their needs. Moreover, we believe that these results will assist patient organizations in having a more proactive role to help researchers accommodate the specific needs of these two groups.

#### 4.3.3. Group-Specific Moderators of Research Participation

Analyzing what factors influence research participation according to HD status, we found that individuals with HDRisk identify more barriers and the need for more support to engage in research than individuals with PreHD. Bardakjian et al. [41] already showed that individuals with PreHD are more willing than other HD groups to enroll in clinical trials regardless of the study design, support received, or therapeutic goals. Interestingly, the obstacles identified by the HDRisk group may be related to the age differences found between the two groups. Having to find someone to take care of the sick parent, having to skip workdays, or having to rethink family planning are concerning issues to the younger members of HD families, particularly individuals at risk for HD [52,53,54]. Additionally, many of the barriers selected by the HDRisk group overlap with those identified by adult caregivers of parents with AD [51], namely, working full-time, having young families, and not having the schedule flexibility to participate in 9-to-5 clinical studies. Thus, this seems to be a common theme across different pathologies. The group-specific moderators of research participation demonstrated once more the urge to design and implement tailored actions to reach individuals with HDRisk and PreHD in a personalized manner and get them more involved in studies and trials. 

### 4.4. Limitations

Some limitations should be considered when interpreting our results.

First, the EHA acknowledges the constraints of using self-report measures to study the perceptions and experiences of clinical populations that display lack of insight regarding symptom presentation and disease status, such as HD-affected individuals [55,56,57]. However, most of the studies that found altered awareness in HD included patients in more advanced disease stages and/or who already presented pronounced cognitive deficits (e.g., [55,56]), which contrasts with the allegedly prediagnostic/presymptomatic status of our study participants. Additionally, as the main goal of this work was to capture the diversity of perceptions and experiences regarding research participation among the HDRisk and PreHD communities across Europe, we believe that having a less stringent assessment method, such as a survey, helped us depict this heterogeneity and gave a more realistic view of how this topic is experienced by these groups.

Second, we did not control for the current medication influence on our work. Indeed, HD drugs reportedly interfere with motor function and cognition [58], which can affect the ability to accurately inform about one’s own condition. Nevertheless, both HDRisk and PreHD individuals typically do not experience obvious clinical symptoms [23,31] and have little contact with the healthcare system, which might indicate that most of our study participants were off any kind of medication. Moreover, our main goal was to capture the opinion of these groups about hypothetical research participation, and we believe that this could be made despite their current medication, unlike, for instance, performing high-precision motor tests [59]. Lastly, as collecting reliable information about medication is a time-consuming and complex process and we wanted to keep the survey completion time relatively short so as to increase the study adherence, we decided to assemble only basic demographic data of survey respondents.

Furthermore, although we were able to gather data from a significant number of individuals with HDRisk and PreHD, studies with larger sample sizes are needed to confirm and expand the current findings.

## 5. Conclusions

This study provides a significant contribution to understanding research participation in HD and it is the first to focus exclusively on the research perceptions and experiences of individuals in a preclinical HD stage. We demonstrated that individuals at risk for HD and with premanifest HD report high motivation to engage in studies and trials, even though they report having limited research experience and literacy. This motivation seems to be influenced by both personal or subjective factors, such as psychological, familial, and financial stability, and interpersonal or objective factors, such as access and support from health professionals. Importantly, we proved that the willingness to participate in research varies according to HD status; individuals with HDRisk expressed more concerns and indicate more barriers to study participation than individuals with PreHD, who were motivated to take part in studies independently of the support received or the study methodology. Lastly, we showed that patient organizations are consistently identified as trusted sources of information about studies and trials, which validates the key role of HD associations in fostering research engagement through tailored education and support of different HD groups. Furthermore, our findings reinforce the relevance of implementing the project Moving Forward in distinct European countries to help bring those traditionally less involved in research closer to study sites. 

## Figures and Tables

**Table 1:** Demographic features of survey respondents.

Demographics				
	Total n = 525	HDRisk n = 263	PreHD n = 262	Chi-Square HDRisk vs. PreHD
	%	%	%	χ^2^ (*p*-value)
**Gender**				
Female	71.6	73.4	69.8	0.808 (0.369)
Male	28.4	26.6	30.2	
Other	0	0	0	-
**Age Interval (years)**				
18 to 24	8.6	12.5	4.6	10.631 (0.001) **
25 to 34	29.5	27.8	31.3	0.791 (0.374)
35 to 44	33.1	35	31.3	0.804 (0.370)
45 to 54	18.3	18.3	18.3	0.000 (0.984)
55 to 64	8.4	5.7	11.1	4.920 (0.027) *
65 to 74	1.5	0.8	2.3	2.046 (0.153)
75 or older	0.6	0	1.1	3.029 (0.082)
**Education Level**				
Did not attend school	0	0	0	-
Completed primary education	1	0.4	1.5	1.829 (0.176)
Completed secondary education	20.4	18.3	22.5	1.473 (0.225)
Graduated from high school	28.4	27.8	29	0.101 (0.751)
Graduated from college	26.5	29.7	23.3	2.740 (0.098)
Completed graduate school	23.8	24	23.7	0.006 (0.938)
**Previous HD Research Experience**				
Yes	31.2	16.3	46.2	54.384 (<0.001) **
No	68.8	83.7	53.8	

HDRisk—individuals at risk for HD whose genetic status is unknown; PreHD—individuals that tested positive for HD and are in a so-called premanifest or prodromal disease stage * Statistically significant at *p ≤* 0.05; ** statistically significant at *p ≤* 0.01.

**Table 2:** Level of knowledge and sources of information about HD research.

Knowledge and Information about HD Research				
	**Total** **n = 525**	**HDRisk** **n = 263**	**PreHD** **n = 262**	**Chi-Square** **HDRisk vs. PreHD**
	%	%	%	χ^2^ (*p*-value)
**Knowledge about HD Research**				
Not good	5.3	6.5	4.2	1.334 (0.248)
Should be better	33.3	39.2	27.5	8.061 (0.005) **
Satisfactory	24.6	26.2	22.9	0.788 (0.375)
Good	28.6	23.2	34	7.467 (0.006) **
Excellent	7.8	4.9	10.7	6.015 (0.014) *
Do not want to know about HD research	0.4	0	0.8	2.015 (0.156)
**Sources of Information about HD Research**				
Internet	78.5	78.7	78.2	0.017 (0.897)
Television	4	2.7	5.3	2.458 (0.117)
Press/newsletters/flyers/booklets	19.8	22.4	17.2	2.284 (0.131)
HD associations and/or support groups	52	51	53.1	0.233 (0.630)
Healthcare professionals	25.5	23.2	27.9	1.505 (0.220)
Family members	25.3	30.8	19.8	8.321 (0.004) **
Not interested in HD research information	0.4	0.4	0.4	0.000 (0.998)

* Statistically significant at *p ≤* 0.05; ** statistically significant at *p ≤* 0.01.

**Table 3:** Factors influencing HD research participation.

Factors Influencing Research Participation				
	**Total** **n = 525**	**HDRisk** **n = 263**	**PreHD** **n = 262**	**Chi-Square** **HDRisk vs. PreHD**
	%	%	%	χ^2^ (*p*-value)
**Factors Preventing Participation**				
I would have to travel to the study site	29.9	30.8	29	0.201 (0.654)
I would not have family support	5.5	5.7	5.3	0.033 (0.857)
I would have personal expenses	33.1	34.6	31.7	0.506 (0.477)
I would have to do motor exams	3.2	4.6	1.9	2.951 (0.086)
I would have to find someone to take care of my sick parent	7	11	3.1	12.737 (<0.001) **
I would have to skip workdays/hours	30.3	36.1	24.4	8.501 (0.004) **
I would have to be involved in the study for a long time	14.1	19	9.2	10.519 (0.001) **
I would have to go through invasive procedures	31.4	36.9	26	7.273 (0.007) **
I would have to do cognitive assessments	4	5.7	2.3	3.982 (0.046) *
I would have to find someone to take care of my children	11.8	11.4	12.2	0.082 (0.775)
I would have to rethink my decisions about family planning	11.2	17.5	5	20.653 (<0.001) **
None of the above	28.6	22.8	34.4	8.561 (0.003) **
**Factors Facilitating Participation**				
I would have assistance from a patient advocate	25.7	26.2	25.2	0.075 (0.784)
I would have family support	26.9	25.9	27.9	0.269 (0.604)
The researcher is my doctor/my family member doctor	17.1	17.5	16.8	0.045 (0.832)
My personal expenses would be reimbursed	42.5	41.1	43.9	0.430 (0.512)
I would get the chance to meet other HD families	27	27	27.1	0.001 (0.979)
I would gain easier access to treatments and health professionals	61.7	62.7	60.7	0.234 (0.629)
I would have psychological and social care available	50.7	55.5	45.8	4.953 (0.026) *
I would not have to go through invasive procedures	26.5	31.6	21.4	6.994 (0.008) **
I know and trust the study team	36.8	35.7	37.8	0.236 (0.627)
I would have regular feedback about my health condition	55.4	53.6	57.3	0.704 (0.402)
None of the above	7	6.5	7.6	0.274 (0.601)

* Statistically significant at *p ≤* 0.05; ** statistically significant at *p ≤* 0.01.

## Data Availability

The datasets generated for this study are available and may be requested from the corresponding author with the permission of the European Huntington Association.

## Supplementary Materials

The following are available online at https://www.mdpi.com/article/10.3390/jpm11080815/s1, Supplementary S1: Survey about the Perceptions and Experiences of Research Participation in the HD Community. Supplementary S2: Information about the Survey.
