# Supplementary material for: Perceptions about Research Participation among Individuals at Risk and Individuals with Premanifest Huntington’s Disease: A Survey Conducted by the European Huntington Association
Source: J Pers Med. 2021 Aug 20;11(8):815. doi: 10.3390/jpm11080815 (PMC8400079; doi:10.3390/jpm11080815)
Supplement: Supplementary file 1 [file jpm-11-00815-s001.zip › Appendix 1_Survey_MovingForward_JPM.pdf]

Survey about the Perceptions and Experiences of Research Participation in the HD Community

*** 1. In what country do you live?**

*** 2. What is your gender?**

- Female
- Male
- Other

*** 3. What is your age?**

- 18 to 24
- 25 to 34
- 35 to 44
- 45 to 54
- 55 to 64
- 65 to 74
- 75 or older

*** 4. What is the highest level of education you have completed?**

*** 5. What is your status regarding Huntington's Disease (HD)?**

- ◯ At risk of Huntington's Disease (You do not know if you carry the genetic mutation of Huntington's Disease and are at risk for inheriting the disease)
- ◯ Premanifest/Prodromal Stage of Huntington's Disease (You had a positive result in the genetic test for Huntington's Disease and do not show symptoms of the disease)

*** 6. Have you ever participated in research studies related to Huntington's Disease?**

- ◯ Yes
- ◯ No

*** 7. How do you rate your knowledge about research studies in Huntington's Disease?**

- ◯ Not good
- ◯ Should be better
- ◯ Satisfactory
- ◯ Good
- ◯ Excellent
- ◯ I do not want to know about HD research

* 8. What are the main sources for you to get information about research studies in Huntington's Disease?

- [ ] Internet
- [ ] Television
- [ ] Press/Newsletters/Flyers/Booklets
- [ ] International/National/Local HD Associations and/or Support Groups
- [ ] Health Care Professionals
- [ ] Family Members
- [ ] I am not interested in HD research information

Is there anything you want to add?

* 9. How important are the following reasons for getting involved in research studies in Huntington’s Disease?

	Not Important	Slightly Important	Moderately Important	Important	Very Important
I will benefit from the best treatments	◯	◯	◯	◯	◯
I will help to produce generalizable knowledge about HD	◯	◯	◯	◯	◯
My doctor thinks it is a good idea	◯	◯	◯	◯	◯
I will help to benefit future generations	◯	◯	◯	◯	◯
My family thinks it is a good idea	◯	◯	◯	◯	◯
I will help others	◯	◯	◯	◯	◯
I know other people that also participate in HD research	◯	◯	◯	◯	◯
I will gain easier access to health professionals and HD experts	◯	◯	◯	◯	◯
I have few therapeutic options	◯	◯	◯	◯	◯
I think it is interesting	◯	◯	◯	◯	◯

Is there anything you want to add?

*** 10. How important are the following reasons for not getting involved in research studies in Huntington's Disease?**

	Not Important	Slightly Important	Moderately Important	Important	Very Important
I do not want to get false hopes	◯	◯	◯	◯	◯
I do not think I can be of any help	◯	◯	◯	◯	◯
I do not think there will be any drugs available in my life time	◯	◯	◯	◯	◯
I do not want to be reminded about the future progression of my disease	◯	◯	◯	◯	◯
I do not have time in my life right now	◯	◯	◯	◯	◯
I do not trust the research team	◯	◯	◯	◯	◯
I do not want to go to clinical units	◯	◯	◯	◯	◯
I do not want to be examined	◯	◯	◯	◯	◯
I do not want to take drugs right now	◯	◯	◯	◯	◯
I do not feel emotionally stable	◯	◯	◯	◯	◯
I do not want to be seen as a "guinea pig"	◯	◯	◯	◯	◯
I fear that my personal data will not be treated as confidential information	◯	◯	◯	◯	◯

Is there anything you want to add?

*** 11. Which of the following factors would prevent you from participating in research studies in Huntington's Disease?**

- [ ] I would have to travel to the study site
- [ ] I would not have family support
- [ ] I would have personal expenses
- [ ] I would have to do motor exams
- [ ] I would have to find someone to take care of my sick parent
- [ ] I would have to skip work days/hours
- [ ] I would have to be involved in the study for a long time
- [ ] I would have to go through invasive procedures (e.g., lumbar puncture or surgery)
- [ ] I would have to do cognitive assessments
- [ ] I would have to find someone to take care of my children
- [ ] I would have to rethink my decisions about family planning
- [ ] None of the above

Is there anything you want to add?

*** 12. Which of the following factors would facilitate your participation in research studies in Huntington's Disease?**

- [ ] I would have assistance from a patient advocate
- [ ] I would have family support
- [ ] The researcher is my doctor/my family member doctor
- [ ] My personal expenses would be reimbursed
- [ ] I would get the chance to meet other HD families
- [ ] I would gain easier access to treatments, health professionals and HD experts
- [ ] I would have psychological and social care available
- [ ] I would not have to go through invasive procedures (e.g., lumbar puncture or surgery)
- [ ] I know and trust the study team
- [ ] I would have regular feedback about my health condition
- [ ] None of the above

Is there anything you want to add?
