# Supplementary material for: Perceptions about Research Participation among Individuals at Risk and Individuals with Premanifest Huntington’s Disease: A Survey Conducted by the European Huntington Association
Source: J Pers Med. 2021 Aug 20;11(8):815. doi: 10.3390/jpm11080815 (PMC8400079; doi:10.3390/jpm11080815)
Supplement: Supplementary file 1 [file jpm-11-00815-s001.zip › Appendix 2_Survey Information_MovingForward_JPM.pdf]

Appendix 2- Information about the Survey

The European Huntington Association (EHA) has just launched a new Project called "Moving Forward" to mobilize two specific groups within HD families to become more involved in research and trials: people at risk for HD and people that tested positive for HD but do not show symptoms (premanifest HD stage).

Right now, we want to understand what the needs, worries and wishes of these two groups are, so that we can plan the best strategies to address them and propose any necessary changes.

To that end, the EHA has created an online Survey that is completely anonymous, easy to fill and extremely brief – it will only take 6-8 minutes to complete.

We kindly ask all the people at risk for HD and people that tested positive for HD but do not show symptoms to fill it, as your first-hand perceptions and experiences of research participation will guide the planning of our work and make it much more meaningful.

Please chose your language from the list, click on the link, and fill the Survey.
